# Genome-Wide Characterization of the Heat Shock Transcription Factor Gene Family in *Begonia semperflorens* Reveals Promising Candidates for Heat Tolerance

**DOI:** 10.3390/cimb47060398

**Published:** 2025-05-27

**Authors:** Zhirou Liu, Nan Lin, Qirui Wang, Enkai Xu, Kaiming Zhang

**Affiliations:** 1 College of Landscape Architecture and Art, Henan Agricultural University, Zhengzhou 450002, China; liuzhirou@126.com (Z.L.);; 2 College of Life Sciences, Henan Agricultural University, Zhengzhou 450046, China

**Keywords:** *Begonia semperflorens*, *HSF* gene family, bioinformatics, expression pattern analysis, transcription factor

## Abstract

*Begonia semperflorens* (*B. semperflorens*) is a popular ornamental plant widely used in landscapes such as plazas and flower beds, and it is also commonly grown as a potted plant indoors. It is known for its adaptability to high temperatures, drought, and shade. Under heat-tolerant conditions, heat shock transcription factors (HSFs) are key transcriptional regulatory proteins that play crucial roles in cellular processes. Despite extensive studies on the *HSF* family in various species, there has been no specific analysis targeting *B. semperflorens*. In this study, we identified 37 members of the *BsHSF* gene family in *B. semperflorens* based on its genome scaffold, which are unevenly distributed across the genome. Phylogenetic analysis reveals that these 37 members can be divided into three subfamilies. Analysis of their physicochemical properties shows significant diversity among these proteins. Except for the BsHSFB7 protein located in the cytoplasm, all other BsHSF proteins were found to be nuclear-localized. A comparison of the amino acid sequences indicates that all BsHSF proteins contain a conserved DNA-binding domain structure. Analysis of the promoter cis-acting elements also suggests that *BsHSFs* may be associated with heat stress and plant secondary metabolism. We further investigated the duplication events of *BsHSF* genes and their collinearity with genes from other Begonia species. Finally, through real-time quantitative PCR, we examined the expression patterns of the 37 *BsHSFs* in different plant tissues (roots, stems, leaves, and flowers) and their expression levels under heat stress treatment. The results show that, except for *BsHSF29*, all *BsHSFs* were expressed in various tissues, with varying expression levels across tissues. Except for *BsHSF33* and *BsHSF34*, the expression levels of almost all *BsHSF* genes increased in response to heat treatment. In summary, these findings provide a better understanding of the role and regulatory mechanisms of HSFs in the heat stress response of *B. semperflorens* and lay the foundation for further exploration of the biological functions of *BsHSFs* in the stress responses of *B. semperflorens*.

## 1. Introduction

*Begonia semperflorens* (*B. semperflorens*) is a widely cultivated ornamental plant with a broad economic market [1]. It is popular due to its diverse flower colors, year-round blooming characteristics, and adaptability to the environment [2]. However, with the rise in global temperatures, its production and application are being challenged, which threatens its production and ornamental value [3]. Heat stress can disrupt its cellular homeostasis, affecting its photosynthesis, cell membrane integrity, and metabolic processes, ultimately impacting its growth and flowering [4]. To address this issue, it is crucial to understand the molecular mechanisms of its heat tolerance. Transcription factors, as the main regulators of gene networks, play a key role in regulating stress responses to various adverse environments [5].

The heat shock transcription (HSF) gene family is a key transcription factor family that responds to heat stress and plays an important role in thermotolerance [6,7,8]. Previous studies have indicated that *HSF* family genes contain several conserved domains [9]. There is a DNA-binding domain (DBD) at the terminus, which can recognize the promoter elements in heat-responsive genes. An adjacent oligomerization domain (OD or HR-A/B), primarily composed of hydrophobic heptapeptide repeats, has been found in all HSF family genes [10,11]. The number of *HSF* gene family members varies among different plants, with 19 in *Rosa chinensis* [12], 21 in *Betula platyphylla* [13], 49 in *Salvia rosmarinus* [14], and 24 in *Ammopiptanthus mongolicus* [15]. Overall, higher plants generally possess more *HSF* family genes than lower plants. This may be attributed to multiple whole-genome duplication (WGD) or whole-genome triplication (WGT) events in plants, especially in higher plants [16]. Members of the HSF gene family respond to a variety of stress conditions [17,18,19]. Previous research has shown that in maize [20], *Hypericum perforatum* [21], and *Eucommia ulmoides* [22], members of the HSF gene family exhibit robust responses to high-temperature stress. Therefore, studying the HSF gene family in *B. semperflorens* is of great significance.

In this study, we systematically identified 37 HSF genes from the genome of *B. semperflorens* and conducted a comprehensive analysis of this gene family using bioinformatics methods. Given that research on the HSF gene in *Arabidopsis thaliana* (*A. thaliana*) is relatively comprehensive, we constructed a phylogenetic tree using the HSF genes from *A. thaliana* and *B. semperflorens* to infer the functions of the HSF gene in *B. semperflorens*. We further explored the heat stress response of *BsHSF* genes and identified a set of candidate genes that potentially play roles in thermotolerance. These findings are crucial for determining the key regulatory factors of stress resistance in *B. semperflorens*. Our results contribute to a deeper understanding of the evolutionary relationships and functional characteristics of the *B. semperflorens HSF* gene family and provide valuable insights for future research on the roles of HSF proteins in stress responses and stress-resistant breeding programs.

## 2. Materials and Methods

### 2.1. Identification of Gene Family Members

The genome data of *A. thaliana* were obtained from the *A. thaliana* Genome Database TAIR (https://www.arabidopsis.org/, accessed on 1 April 2025) [23]. The protein sequences of the *HSF* family members of *A. thaliana* were used as seed sequences to perform a BLAST (2.13.0) alignment against the proteome file of *B. semperflorens*, with an E-value of 1 × 10^−5^. Sequences with homology greater than 50% were selected, and duplicates were removed to obtain the candidate sequences. Subsequently, the HMM model of HSF proteins (PF00447) was downloaded from the Pfam database [24], and hmmsearch from HMMER 3.0 [25] was used to screen the HSF protein members of *B. semperflorens*, with an E-value of 1 × 10^−10^, and the proteome file of *B. semperflorens* set as the protein database. The candidate protein members obtained by the two methods were compared, and the common sequences were selected while removing duplicates. Finally, the protein members were verified by their domain structure using the CD-search tool from NCBI(https://www.ncbi.nlm.nih.gov/cdd, accessed on 1 April 2025) [26], with an E-value of 1 × 10^−5^. Sequences containing at least one complete HSF_DNA-bind domain were selected. The resulting protein members were identified as the HSF protein family members of *B. semperflorens*.

### 2.2. Localization and Numbering of BsHSFs

Based on the genome annotation file and the identified *BsHSF* gene family members, only scaffolds containing *BsHSF* genes were selected for display due to the lack of chromosome data. The localization of *BsHSF* genes was visualized using TBtools v.2.069 [27], and they were numbered according to their order on the scaffolds.

### 2.3. Physicochemical Properties

The physicochemical properties of the 37 BsHSF sequences were analyzed using ExPASy (https://www.expasy.org/, accessed on 1 April 2025) [28]. The subcellular localization of the proteins was predicted using WoLF PSORT (https://wolfpsort.hgc.jp/?utm_source=chatgpt.com, accessed on 1 April 2025) [29].

### 2.4. Phylogenetic Tree Construction

The phylogenetic tree of HSF proteins from *B. semperflorens* and *A. thaliana* was constructed using MEGA-X 11.0 software [30]. Sequence alignment was performed using ClustalW, and the neighbor-joining method was employed for tree construction. The bootstrap method was used for testing, with the bootstrap value set to 1000, while other parameters were kept at their default settings. Additionally, an intraspecific phylogenetic tree for the HSF proteins of *B. semperflorens* was constructed using MEGA-X 11.0 software. Sequence alignment was performed using MUSCLE v5.1, and the neighbor-joining method was employed for tree construction. The bootstrap method was used for testing, with the bootstrap value set to 1000, while other parameters were kept at their default settings. The phylogenetic trees were visually enhanced using iTOL (https://itol.embl.de/, accessed on 1 April 2025) [31] and Adobe Illustrator 2022 software.

### 2.5. Collinearity Analysis

The genome file and gene annotation file of *Begonia darthvaderiana* (*B. darthvaderiana*) were downloaded from NCBI. The interspecific collinearity analysis between *B. semperflorens* and *B. darthvaderiana* was conducted using the One step MCScanX tool in TBtools-II v2.210 software. In addition, the intraspecific collinearity analysis of Begonia was performed using the One step MCScanX tool in TBtools-II v2.210 software. The images were then refined using Adobe Illustrator 2022 software.

### 2.6. Gene Structure Analysis

The annotation information of the *BsHSF* genes was extracted using TBtools-II v2.210. The gene structure analysis was conducted via the GSDS website (http://gsds.cbi.pku.edu.cn/, accessed on 1 April 2025) [32]. The images were then adjusted using Adobe Illustrator 2022 software.

### 2.7. Conserved Motif and Domain Analysis

The conserved motif analysis of the BsHSF protein was performed using the MEME online tool (https://meme-suite.org/meme/, accessed on 1 April 2025) [33], with the maximum number of motifs set to 10 and other settings kept as default. The conserved domain analysis was conducted using the NCBI website, with an E-value of 1 × 10^−5^. The analysis results of motifs and domains were visualized using TBtools-II v2.210 software.

### 2.8. Cis-Acting Element Analysis in the Promoter Region

The sequence of 2000 bp upstream of the *BsHSF* gene promoter was extracted using TBtools-II v2.210. The prediction of cis-acting elements in the promoter region was performed using the PlantCARE website (https://bioinformatics.psb.ugent.be/webtools/plantcare/html/, accessed on 2 April 2025) [34]. The prediction results were visualized using TBtools-II v2.210. The images were then refined using Adobe Illustrator 2022 software.

### 2.9. Plant Materials and Treatment

**Figure 1:**
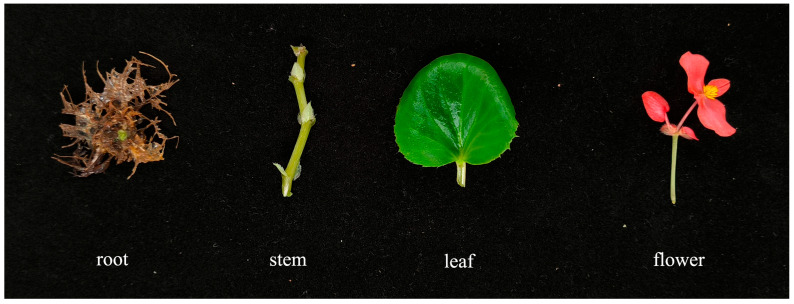
Sampling tissues of *B. semperflorens* for quantitative real-time PCR.

The required *B. semperflorens* ‘*Super Olympia*’ plants were cultivated at the College of Landscape Architecture and Art, Henan Agricultural University. The photoperiod was set at 12 h of light and 12 h of darkness, with a light intensity of 200 µmol/m^2^/s and a cultivation temperature of 25 °C. Plants were planted in sterile bottles and placed in a growth chamber. Healthy and uniformly sized *B. semperflorens* plants, aged 3 to 4 months and grown in sterile bottles, were selected for heat stress treatment. Tissues of roots, stems, leaves, and flowers (Figure 1) were collected before treatment for the detection of tissue-specific expression. Other cultivation conditions remained unchanged, while the cultivation temperature was set at 38 °C, slightly lower than its high-temperature semi-lethal temperature. Samples were collected from the treated plants at 0 h, 12 h, 24 h, 36 h, and 48 h after treatment. Each sample weighed 0.1 g and was placed into a 2 mL centrifuge tube, then quickly frozen in liquid nitrogen and stored in a −80 °C freezer for later use. Each treatment had three biological replicates, with each replicate randomly selecting different 9 *B. semperflorens* plants.

### 2.10. RNA Extraction and Real-Time Quantitative PCR Analysis

Total RNA was extracted from each tissue sample using the PureTotal RNA Extraction Kit (DP441) designed for polysaccharide–polyphenol plants. The RNA was then reverse-transcribed into cDNA using the HiScript III 1st Strand cDNA Synthesis Kit (Nanjing Vazyme Biotech Co., Ltd., Nanjing, China) with the gDNA Eraser. Primers for RT-qPCR were synthesized by Sangon Biotech, and all primer sequences are listed in Table S1. The *Bs18s* gene was used as the housekeeping gene [35]. The reaction system and program were configured according to the instructions of SYBR Premix Ex Taq™ II (TaKaRa BIO Inc., Beijing, China). The data were analyzed using the 2^−ΔΔCT^ method. One-way analysis of variance (ANOVA) was performed using SPSS 22.0 software, followed by Duncan’s multiple comparison test. GraphPad Prism 10.0 [36] was used for the graphical representation of the data.

## 3. Results

### 3.1. Identification and Physicochemical Characterization of the BsHSF Gene Family

After removing redundant sequences, we identified 37 HSF genes from the *B. semperflorens* genome using bioinformatics methods. These genes were named *BsHSF1* to *BsHSF37* based on their genomic locations. Table 1 lists the detailed information, including the physicochemical properties of the 37 BsHSF proteins. The lengths of the BsHSF proteins range from 81 amino acids (BsHSF7) to 521 amino acids (BsHSF17), with molecular weights varying from 9.62 kDa (BsHSF7) to 57.48 kDa (BsHSF17). The theoretical isoelectric points (pI) range from 4.63 (BsHSF11) to 9.86 (BsHSF7). Notably, with the exception of BsHSF7, BsHSF15, BsHSF3, BsHSF25, BsHSF2, BsHSF32, and BsHSF33, all BsHSF proteins are classified as acidic (pI < 7). The average hydrophilicity value of the BsHSF proteins is negative, indicating that they are predominantly hydrophilic. Moreover, the average instability index of these proteins is 56.7 (above the threshold of 40), suggesting that they are likely to be unstable. Subcellular localization prediction shows that, except for BsHSF7, which is localized in the cytoplasm, all other BpHSF proteins are located in the nucleus.

### 3.2. Localization of BsHSF Genes

**Figure 2:**
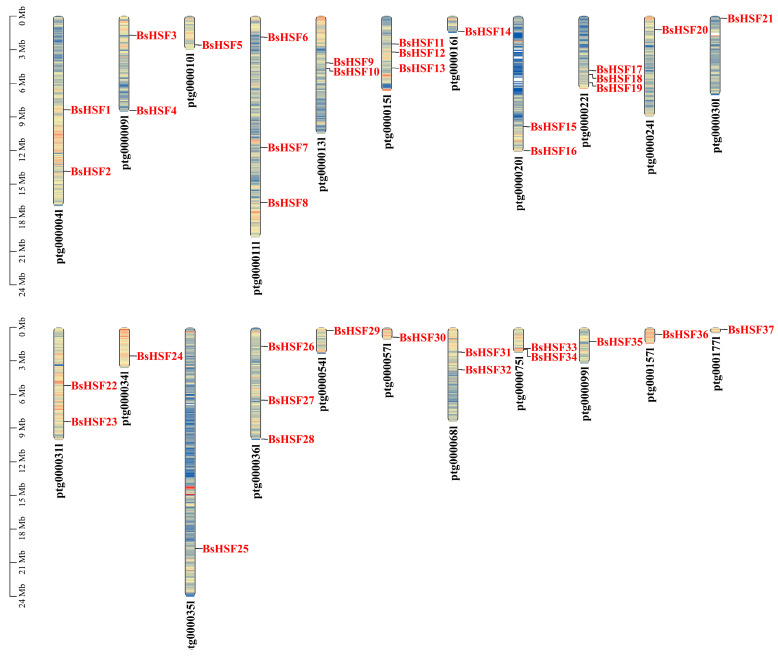
Localization of the *BsHSF* genes. Scale bars are in Mb, and scaffolds numbers are shown above the corresponding chromosomes.

The localization analysis indicates that, despite the lack of chromosomal data, the distribution pattern of the 37 *BsHSF* genes is not uniform (Figure 2). Among the 71 scaffolds, only 22 contain *BsHSF* genes. The longest scaffold, ptg000035l, with a length of 24 MB, harbors only one *BsHSF* gene, *BsHSF25*. In contrast, three genes are found on scaffolds ptg000011l (19.7 MB), ptg000015l (6.7 MB), ptg000022l (6.5 MB), and ptg000036l (10.0 MB). Specifically, *BsHSF6*, *BsHSF7*, and *BsHSF8* are located on ptg000011l; *BsHSF11*, *BsHSF12*, and *BsHSF13* are on ptg000015l; *BsHSF17*, *BsHSF18*, and *BsHSF19* are on ptg000022l; and *BsHSF26*, *BsHSF27*, and *BsHSF28* are on ptg000036l.

### 3.3. Phylogenetic Analysis of BsHSF

**Figure 3:**
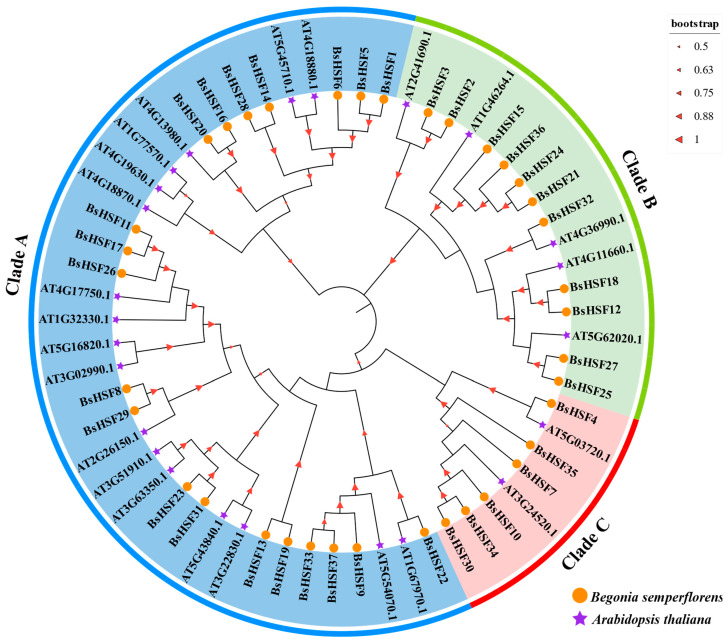
Phylogenetic analysis of *BsHSF* genes was conducted using HSF sequences from *B. semperflorens* (BsHSF) and Arabidopsis thaliana (AT). As a result, different HSF classes are distinguished in various colors, each representing a distinct class.

A neighbor-joining phylogenetic tree was constructed to analyze the evolutionary relationships of *HSF* genes from *B. semperflorens* and *Arabidopsis thaliana* (Figure 3). This analysis included 61 protein sequences: 37 from *B. semperflorens* and 24 from *Arabidopsis thaliana*. Based on the classification system of the HSF protein family in the model plant *Arabidopsis thaliana*, the *BpHSF* genes were divided into three major groups corresponding to three subfamilies (A, B, and C). In *B. semperflorens*, subfamily A contains 20 BsHSF members, subfamily B contains 11 members, and subfamily C contains 6 members. In *Arabidopsis thaliana*, subfamily A contains 17 BsHSF members, subfamily B contains 5 members, and subfamily C contains 2 members. *B. semperflorens* has more members in each subfamily than *Arabidopsis thaliana*, with the greatest increase observed in subfamily C, where the number of members in *B. semperflorens* is three times that of *Arabidopsis thaliana*.

### 3.4. Protein Motifs and Gene Structure of BsHSF Genes

**Figure 4:**
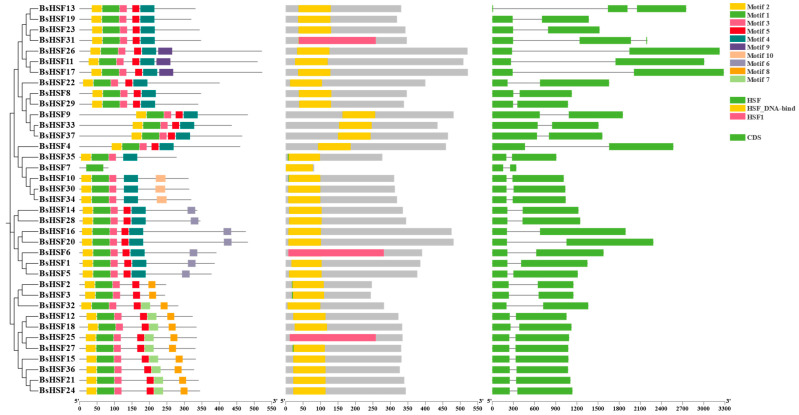
Gene structure and conserved motifs analysis of BsHSFs: phylogenetic relationship tree; conserved motifs of BsHSFs; SMART conserved HSF domain; gene structure of BsHSFs.

Analysis of gene structure and conserved motifs can provide deeper insights into gene function and evolution. Using the MEME tool, we identified 10 conserved motifs containing 20 to 50 amino acids in the BsHSF family, with variable distribution among different BsHSFs. The number of conserved motifs ranges from one to six, with BsHSF7 containing only one motif, while most BsHSFs contain 5–6 motifs (Figure 4). The differences in the number and distribution of motifs among family members suggest that different BsHSFs may have distinct biological functions. All BsHSF proteins contain Motif 1, indicating that Motif 1 is highly conserved in BsHSFs and may play an important role. Except for BsHSF7, all BsHSF proteins contain Motif 2 and Motif 3, while Motif 8 is only present in members of subfamily B. Except for BsHSF13 and BsHSF31, which have three exons, all other BsHSFs have two exons. This indicates that the evolution of BsHSFs is more conservative compared to other species.

### 3.5. Analysis of Cis-Acting Elements in the Promoters of BsHSF Genes

**Figure 5:**
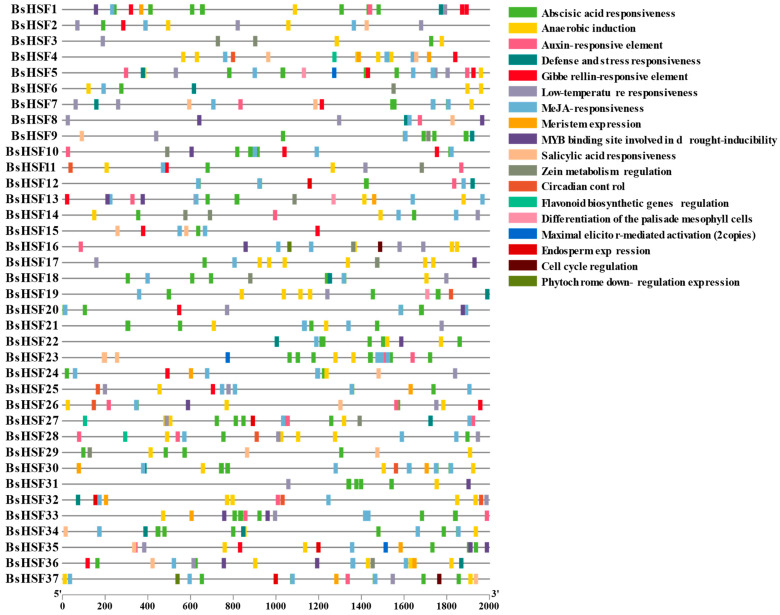
The distribution of cis-regulating elements in the promoter region of the HSF gene in *B. semperflorens*. Each promoter is located at the 2000 bp upstream region of BsHSF gene. The colored blocks represent 18 kinds of cis-elements.

Transcription factors play a crucial role in mediating plant responses to biotic and abiotic stresses by regulating various cis-regulatory elements in gene promoter sequences. To explore the potential biological functions and regulatory networks of *BsHSF* genes, we analyzed the cis-regulatory elements within the promoter regions of *BsHSF* genes (spanning 2000 bp upstream of the start codon) (Figure 5). A total of 598 cis-regulatory elements were identified in this analysis, with functional descriptions including abscisic acid responsiveness, anaerobic induction, auxin-responsive element, cell cycle regulation, circadian control, defense and stress responsiveness, differentiation of the palisade mesophyll cells, endosperm expression, flavonoid biosynthetic genes regulation, gibberellin-responsive element, low-temperature responsiveness, maximal elicitor-mediated activation, MeJA-responsiveness, meristem expression, the MYB binding site involved in drought-inducibility, phytochrome down-regulation expression, salicylic acid responsiveness, and zein metabolism regulation. Among these, the most abundant elements were abscisic acid responsiveness and MeJA-responsiveness, with 165 and 164 occurrences, respectively, indicating that *BsHSFs* may respond to abscisic acid and MeJA. The presence of other cis-acting elements also suggests that *BsHSFs* may be associated with abiotic stress responses and plant secondary metabolism.

### 3.6. Gene Duplication Events and Collinearity of BsHSF Genes

**Figure 6:**
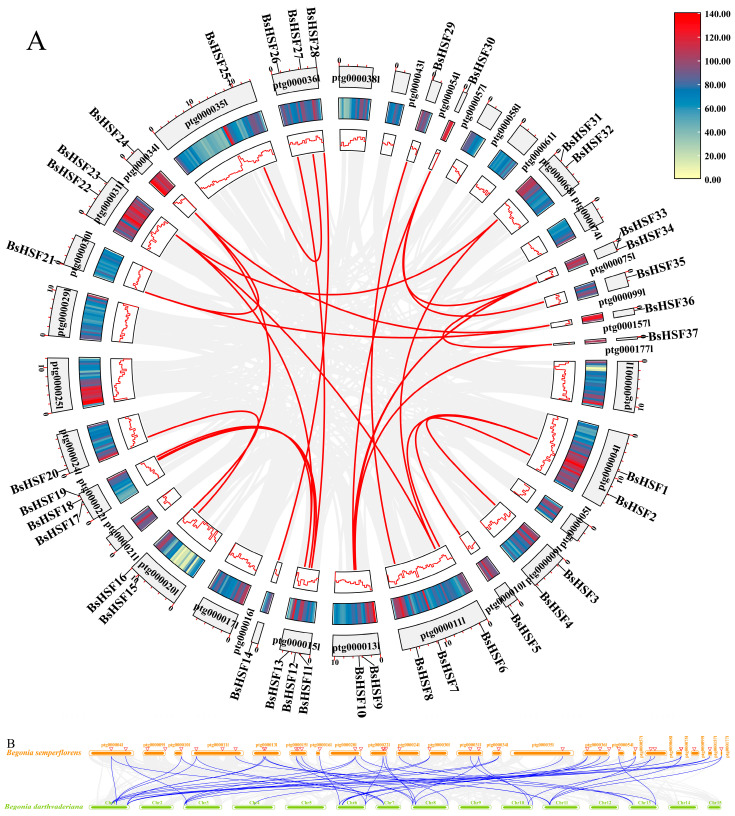
Collinearity of the *HSF* gene family in different species. (**A**) Collinearity of the HSF gene family in *B. semperflorens*; (**B**) Collinearity of the *HSF* gene family between *B. semperflorens* and *B. darthvaderiana*. The heatmaps and line plots illustrate the gene density within each chromosome. The grey curves in the background indicate all segmental duplications (SDs) within the *B. semperflorens* genome and between the genomes of *B. semperflorens* and *B. darthvaderiana.* The blue curves highlight the SDs involving *HSF* genes. Darker regions indicate a higher number of SDs and denser curves, while lighter regions indicate fewer SDs and sparser curves.

Gene duplication is crucial for the evolution of plant genomes. To identify duplication events of *BsHSF* genes, the gene pairs (GPs) were counted, and a homology analysis was conducted within the species (Figure 6A). The results show that 24 segmental duplications (SDs) occurred among the 34 *BsHSF* genes, with no tandem duplications detected. Only three *BsHSF* genes, namely, *BsHSF22*, *BsHSF7*, and *BsHSF4*, did not undergo SDs. The findings indicate that SDs play a key role in the expansion of *BsHSF* genes.

To better elucidate the evolutionary relationships of *B. semperflorens*, an interspecific collinearity map was constructed between *B. semperflorens* and its congener *B. darthvaderiana* (Figure 6B). It was found that 18 genes in *B. darthvaderiana* corresponded to 30 genes in *B. semperflorens*, with 47 gene pairs (GPs) identified between them, indicating strong collinearity. Moreover, most genes in *B. darthvaderiana* corresponded to more than one gene in *B. semperflorens*.

### 3.7. Expression Patterns of BsHSF Genes in Different Tissues

**Figure 7:**
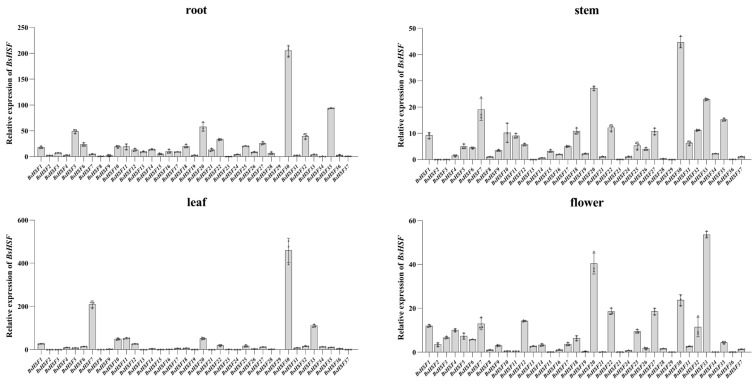
qRT-PCR analysis of *BsHSF* genes in four different tissues.

The expression of *BsHSF* genes in different tissues (root, stem, leaf, and flower) of *B. semperflorens* was detected using quantitative real-time PCR (Figure 7). Except for *BsHSF29*, which showed no detectable expression in root and leaf tissues, all other genes were expressed in each tissue. The expression levels of each gene varied among different tissues. In roots, higher expression levels were observed for *BsHSF5*, *BsHSF20*, *BsHSF22*, *BsHSF30*, *BsHSF32*, and *BsHSF35*. In stems, *BsHSF7*, *BsHSF20*, *BsHSF30*, *BsHSF33*, and *BsHSF35* exhibited higher expression levels. In leaves, the expression levels of the genes varied greatly, with *BsHSF30* showing a significantly higher expression than other genes. *BsHSF1*, *BsHSF7*, *BsHSF10*, *BsHSF11*, *BsHSF12*, *BsHSF20*, *BsHSF30*, and *BsHSF33* also had relatively high expression levels. In flowers, higher expression levels were detected for *BsHSF20*, *BsHSF22*, *BsHSF27*, *BsHSF30*, and *BsHSF33*.

### 3.8. Expression Patterns of BsHSF Genes Under Heat Stress Treatment

**Figure 8:**
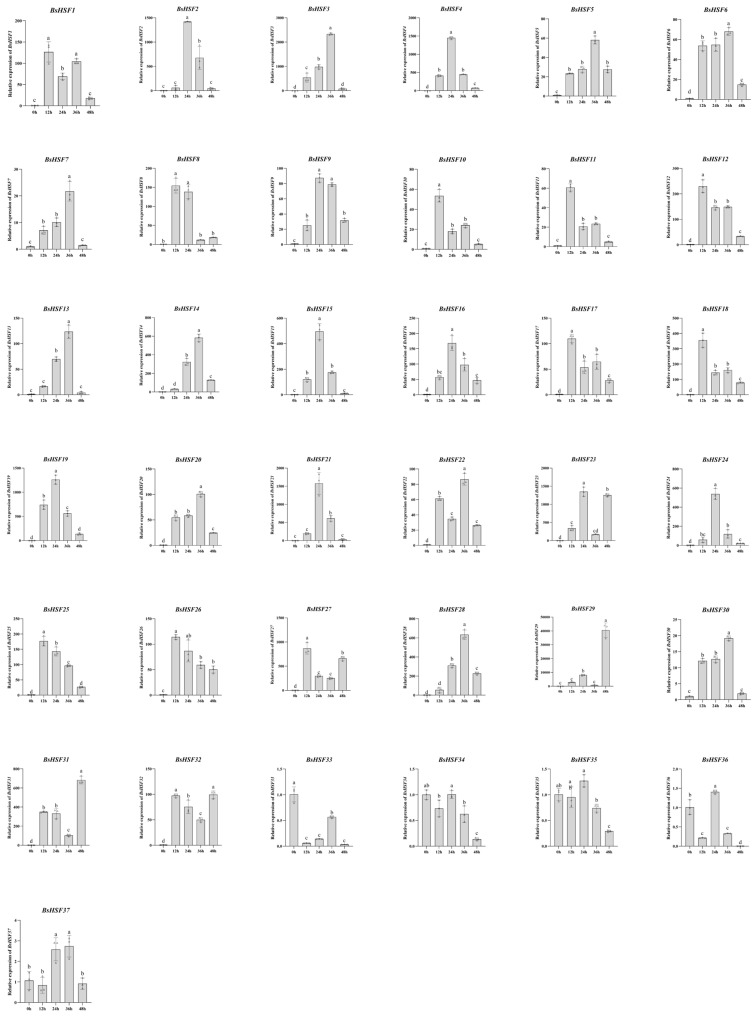
qRT-PCR analysis of BsHSF genes under heat stress. Different lowercase letters above the bars indicate statistically significant differences (*p* < 0.05) using one-way ANOVA.

To investigate the response patterns of *B. semperflorens* to heat stress, the expression of *BsHSF* genes after heat treatment was detected using quantitative real-time PCR (Figure 8). It was found that, overall, almost all *BsHSF* genes, except for *BsHSF33* and *BsHSF34*, showed increased expression levels in response to heat treatment. The expression levels of *BsHSF2*, *BsHSF3*, *BsHSF4*, *BsHSF19*, *BsHSF21*, *BsHSF23*, *BsHSF27*, and *BsHSF29* increased significantly in response to heat treatment. Notably, *BsHSF29*, which is almost undetectable or has extremely low expression under normal temperature conditions, showed an approximately 40,000-fold increase in expression after 48 h of heat treatment compared to 0 h. This indicates that *BsHSF29* strongly responds to heat treatment and may be associated with plant thermotolerance.

## 4. Discussion

*Begonia semperflorens* is widely used for decorating various flower beds and squares due to its characteristic of blooming all year round. It has relatively strong resistance, with features of drought tolerance and high-temperature endurance [37]. Transcription factors play important regulatory roles in plant growth, development, and responses to stress, among which heat shock transcription factors are in a core position [8]. This study identified 37 *HSF* transcription factors, which is more than the number of *HSF* gene family members in *Arabidopsis thaliana* (21 genes), *Oryza sativa* (25 genes), *Solanum lycopersicum* (26 genes), *Brassica oleracea* (35 genes), and *Brassica rapa* (36 genes). Thus, we supposed that more *HSF* transcription factors may be related to the better heat tolerance of *B. semperflorens*. Higher plants have generated more members of the *HSF* gene family through multiple whole-genome duplication (WGD) or whole-genome triplication (WGT) events to adapt to high-temperature environments [38,39,40]. The distribution of these genes in the genome is not uniform, and the length of the segments is not correlated with the number of HSF genes distributed (Figure 2).

In angiosperms, the *HSF* gene family is divided into three subfamilies: A, B, and C (Figure 3). Based on gene structure and phylogenetic relationships, we classified the 37 BsHSF genes into subfamilies A, B, and C. Specifically, subfamily A contains 20 genes, subfamily B contains 11 genes, and subfamily C contains 6 genes. Although the total number is greater than that of the HSF gene family members in *Arabidopsis*, the main increase in members belongs to subfamilies B and C. The number of subfamily A members does not differ significantly. Such changes may also imply the functions of the *HSF* family genes in *B. semperflorens*. Most members of subfamily A have one or more AHA motifs at the C-terminus. These motifs act as transcriptional activators, functioning by binding to heat stress elements and playing a dominant role in the heat stress response [41,42,43,44,45]. Some members of subfamily B contain a tetrapeptide—LFGV—at their C-terminus, which may act as a repressive motif through interaction with an unknown co-repressor [46,47]. There is relatively less research on subfamily C. Studies on grass crops have found that there are multiple *HSFC2* members in rice and wheat. For example, *TaHSFC2a-B* in wheat has been reported to act as a trans-activator of heat shock proteins, thereby positively regulating thermotolerance [48]. In lilies, *LlHSFC2* is induced under heat shock conditions and exhibits the ability to repress the expression of heat protection genes. However, it can also interact with *HSFAs*, acting as a co-activator to activate their transcriptional activity and participate in the establishment of thermotolerance [49]. The increased number of subfamily C members in *B. semperflorens* may imply the existence of a unique heat stress resistance pathway.

Through amino acid sequence alignment analysis, it has been shown that all 37 BsHSF proteins contain the DNA-binding domain (DBD) conserved domain, which is consistent with studies in other plants. The structure of HSF family members is relatively conserved in plants, with most containing a DBD, an oligomeric domain (OD or HR-A/B, hydrophobic heptad repeat region), and a nuclear localization signal (NLS) [10]. In the analysis of the *HSF* promoter, a large number of ABA and MeJA response elements, as well as elements related to abiotic stress responses and plant secondary metabolism, were identified. Considering that transcription factors can exert transcriptional activation or repression by binding to cis-elements in the promoters of related genes, it is hypothesized that some functional genes may regulate gene expression by binding to certain cis-elements in the *BsHSF* promoters. This implies that *BsHSFs* may respond to ABA and MeJA and may be associated with abiotic stress responses and plant secondary metabolism. However, the mining of related genes and the specific regulatory mechanisms require further research in the future. Gene duplication and repetition play important roles in the evolution and expansion of gene families [50]. Studies have pointed out that the *AhHSF* gene family in peanuts has utilized gene duplication and repetition during the evolutionary process [51]. In *B. semperflorens*, a large number of homologous genes have been found in the HSF gene family, and compared with the *HSF* gene family members in the congener *B. darthvaderiana*, there has been an expansion. This indicates that there has been a significant amount of duplication and repetition of *HSF* genes in *B. semperflorens*, which may be related to the stronger resistance of *B. semperflorens*.

We can infer their primary regulatory regions by analyzing the expression patterns of the *HSF* gene family members in different tissues of *B. semperflorens*. We found that the member *BsHSF30*, which belongs to the C subfamily, is highly expressed in all tissues except flowers. This reveals that *BsHSF30* may play a role in plant growth and development. This aligns with previous studies indicating that the HSFC1 subclass, which is unique to dicots, may be involved in the developmental processes of plants [10]. Members of the HSF gene family have also been reported to be associated with root development. The *HSFB4* member of the B subfamily of the *A. thaliana* HSF gene family affects root development. *HSFB4* is expressed in various tissues, with the highest expression level in roots. Overexpression of the *HSFB4* gene in *A. thaliana* leads to rough root epidermis and promotes epidermal splitting, which, in turn, promotes the development of lateral roots [52]. Members of the B subfamily of the HSF gene family, including *BsHSF32*, *BsHSF21*, and *BsHSF18*, are highly expressed in roots. Among them, *BsHSF21* is highly expressed only in roots, while *BsHSF32* and *BsHSF18* also have relatively high expression in stems and flowers. It is speculated that these three genes may be related to the root development of *B. semperflorens*. Except for *BsHSF29*, which was not detected in root and leaf tissues, all other genes were expressed in all tissues. We also detected and analyzed the expression patterns of *BsHSFs* in *B. semperflorens* after heat stress treatment. Almost all *BsHSFs* showed increased expression levels in response to heat stress, with several being particularly notable, including *BsHSF2*, *BsHSF3*, *BsHSF4*, *BsHSF19*, *BsHSF21*, *BsHSF23*, *BsHSF27*, and *BsHSF29.* They are all highly likely to be associated with the heat stress resistance of *B. semperflorens*. Notably, the expression level of *BsHSF29* increased by approximately 40,000 times after 48 h of high-temperature treatment compared to 0 h. As previously mentioned, no expression of *BsHSF29* was detected in root and leaf tissues, suggesting that it may be a gene specifically induced by heat stress treatment. In *Solanum lycopersicum* and *A. thaliana*, the expression of some HSF genes is also inducible. Under normal temperature conditions, they are almost not expressed or their expression levels are maintained at an extremely low level. High temperatures can cause a dramatic increase in their expression levels [18,53]. In evolutionary analysis, *BsHSF29* clustered with *Arabidopsis AT2G26150.1*. Moreover, the overexpression of *Arabidopsis AtHsfA2* (*AT2G26150.1*) can enhance the tolerance of *Arabidopsis* to high-temperature stress [54], implying that *BsHSF29* is likely an important candidate gene for *B. semperflorens* to respond to high-temperature stress.

*HSF* is an important transcription factor that positively regulates the stress resistance of plants. In this study, based on bioinformatics analysis, we found that the expression levels of the *BsHSF* gene vary in different tissues and under heat stress, indicating that their functions in plants are not entirely the same. In the future, knockout and overexpression of the *BsHSF* gene are needed to further identify its molecular functions, which will provide new insights into the molecular regulatory pathways of the physiological mechanisms of *B. semperflorens*. Some members of the *BsHSF* gene family may be valuable candidate genes for the molecular breeding of *B. semperflorens* resistance.

## Figures and Tables

**Table 1:** Physicochemical properties of proteins encoded by the 37 *BsHSF* genes in *B. semperflorens*.

Rename	Gene ID	Number of Amino Acid	Molecular Weight	Theoretical pI	Instability Index	Aliphatic Index	Grand Average of Hydropathicity	Subcellular Localization
*BsHSF1*	g2919.t1	385	44,307.25	5.38	54.6	74.42	−0.794	Nuclear
*BsHSF2*	g4110.t1	246	28,643.14	7.69	49.15	66.59	−0.985	Nuclear
*BsHSF3*	g6017.t1	243	28,082.52	8.62	54.74	60.95	−1.03	Nuclear
*BsHSF4*	g7033.t2	458	52,759.41	5.15	66.23	68.76	−0.689	Nuclear
*BsHSF5*	g7541.t1	376	43,643.24	5.84	50.77	80.61	−0.67	Nuclear
*BsHSF6*	g7952.t1	390	44,297.31	5.43	54.34	73.74	−0.794	Nuclear
*BsHSF7*	g9408.t1	81	9621.97	9.86	32.63	68.64	−0.56	Cytoplasmic
*BsHSF8*	g10132.t1	346	39,200.81	4.8	58.47	76.91	−0.618	Nuclear
*BsHSF9*	g11656.t1	480	53,724.09	5.34	56.47	74.92	−0.614	Nuclear
*BsHSF10*	g11734.t1	310	35,367.96	5.76	61.04	69.13	−0.561	Nuclear
*BsHSF11*	g12866.t1	508	56,333.3	4.63	53.03	67.15	−0.726	Nuclear
*BsHSF12*	g12999.t1	322	35,388.58	5.05	59.62	71.74	−0.541	Nuclear
*BsHSF13*	g13272.t1	330	39,491.62	5.91	58.01	65.55	−0.898	Nuclear
*BsHSF14*	g13857.t1	335	39,352.21	5.61	53.66	64	−0.857	Nuclear
*BsHSF15*	g16136.t1	331	37,427.53	8.8	55.03	71.27	−0.501	Nuclear
*BsHSF16*	g16542.t1	474	53,737.31	5.21	64.51	70.97	−0.826	Nuclear
*BsHSF17*	g17820.t1	521	57,480.9	4.66	63.41	66.76	−0.643	Nuclear
*BsHSF18*	g17877.t2	333	36,687.07	4.97	55.75	78.47	−0.504	Nuclear
*BsHSF19*	g17997.t1	318	37,858.8	5.81	64.24	67.7	−0.846	Nuclear
*BsHSF20*	g18359.t1	480	54,441.75	5.87	62.63	73.52	−0.752	Nuclear
*BsHSF21*	g23282.t1	339	38,820.51	6.53	55.13	66.73	−0.6	Nuclear
*BsHSF22*	g25236.t1	399	45,983.14	4.74	48.14	75.04	−0.584	Nuclear
*BsHSF23*	g25942.t1	342	39,151.85	5.02	68.5	70.41	−0.714	Nuclear
*BsHSF24*	g26841.t1	343	39,072.9	6.73	55	73.03	−0.549	Nuclear
*BsHSF25*	g29399.t1	334	37,450.29	8.14	59.39	71.26	−0.578	Nuclear
*BsHSF26*	g30373.t1	520	57,020.55	5	60.02	67.87	−0.594	Nuclear
*BsHSF27*	g31168.t1	330	36,756.36	6.94	55.84	73.88	−0.534	Nuclear
*BsHSF28*	g31755.t1	344	40,447.32	5.42	54.09	64.88	−0.872	Nuclear
*BsHSF29*	g33546.t1	338	37,620.03	5.52	49.37	70.47	−0.693	Nuclear
*BsHSF30*	g34334.t1	312	35,559.17	5	73.79	68.4	−0.546	Nuclear
*BsHSF31*	g36487.t1	346	39,476.21	5.02	56.16	67.57	−0.804	Nuclear
*BsHSF32*	g36767.t1	281	31,424.19	7.59	43.38	61.42	−0.829	Nuclear
*BsHSF33*	g38782.t1	434	48,995.12	7.11	62.01	65.37	−0.614	Nuclear
*BsHSF34*	g38809.t1	318	36,390.19	5.67	60.89	68.71	−0.559	Nuclear
*BsHSF35*	g39393.t1	276	32,442.08	5.36	58.69	85.76	−0.592	Nuclear
*BsHSF36*	g40203.t1	326	37,186.68	6.46	54.7	67.61	−0.598	Nuclear
*BsHSF37*	g40444.t1	464	51,457.67	5.59	57.65	69.29	−0.486	Nuclear

## Data Availability

The original contributions presented in this study are included in the article/Supplementary Material. Further inquiries can be directed to the corresponding author(s).

## Supplementary Materials

The following supporting information can be downloaded at: https://www.mdpi.com/article/10.3390/cimb47060398/s1.
