# Supplementary material for: Genome-Wide Characterization of the Heat Shock Transcription Factor Gene Family in Begonia semperflorens Reveals Promising Candidates for Heat Tolerance
Source: Curr Issues Mol Biol. 2025 May 27;47(6):398. doi: 10.3390/cimb47060398 (PMC12191710; doi:10.3390/cimb47060398)
Supplement: Supplementary file 1 [file cimb-47-00398-s001.zip › cimb-3623738-supplementary.pdf]

Table S1. Primer Sequences for RT-qPCR Analysis

Primer Name	Sequence
18S-FORWARD	GCTACCACATCCAAGGAAGG
18S-REVERSE	CAATGGATCCTCGTTAAGGG
g2919.t1-FORWARD	CAGGAAGAGGAGGCTACCCAGAACT
g2919.t1-REVERSE	CTAAGTCGATGTTGGAAGTTTGGTG
g4110.t1-FORWARD	GGAAGAGGAGGACGACGAGAG
g4110.t1-REVERSE	ATGATTGAGAGAGTGTAAGGCTTTGG
g6017.t1-FORWARD	CACGAAGACGAAGACGAAGACG
g6017.t1-REVERSE	CTTTGGTCGCTTGCCCTTGTC
g7033.t2-FORWARD	TTAATGGCGGAAGTTGTTGAATTGC
g7033.t2-REVERSE	CATCTGCTTCTGTCTCTTCTCTGC
g7541.t1-FORWARD	AACCAGTCCACAGCCATTCTTTAC
g7541.t1-REVERSE	TTGTCCTGTTTTAGCCCCTCAATATC
g7952.t1-FORWARD	TTGCCAGACTTTAACCACTCCTAAC
g7952.t1-REVERSE	ATCCACCAGCCGCCTCATG
g9408.t1-FORWARD	CTTCGTTCGTCAGCTAAACACTTAC
g9408.t1-REVERSE	CAAACCATGCTTCTGCCCTCTC
g10132.t1-FORWARD	CAGCAGCAGATGTTGTCGTTCC
g10132.t1-REVERSE	AGTCTTCTCTTCCGTCCGATGTC
g11656.t1-FORWARD	GGAGGGCTTACACGAGACAGG
g11656.t1-REVERSE	GTCACGACCTTGATTCCACGATAC
g11734.t1-FORWARD	CGGCGTATTTCAAGCACAACAAC
g11734.t1-REVERSE	TCTGCCCTCGTAGAAACCACTC
g12866.t1-FORWARD	CGGCGTATTTCAAGCACAACAAC
g12866.t1-REVERSE	TCTGCCCTCGTAGAAACCACTC
g12999.t1-FORWARD	GAACGGAACAACAAATCGGACATC
g12999.t1-REVERSE	ACGACGGTTCTGCTCCTCTAC
g13272.t1-FORWARD	CGAGTCAAGGAGGAGGAGGTTC
g13272.t1-REVERSE	GCGGAGGGGCTGGTTCTTC
g13857.t1-FORWARD	AGACCCGTTTGCTTCCATCCTC
g13857.t1-REVERSE	CTTCTTCCCTTCCAACCTCAATCTG
g16136.t1-FORWARD	CTCGTGACCTTCTTCCTAACTACTTC
g16136.t1-REVERSE	CGTTCGCAAACTCCCATCTATCC
g16542.t1-FORWARD	CGTGTAACATGGTGGATGACTCAG
g16542.t1-REVERSE	CTGGAAGAAGAAGCCGAGAGAAC
g17820.t1-FORWARD	AGTCAACCACTACAGCAATCTCAAG
g17820.t1-REVERSE	AGCCTCTCAACCTCCTCTTCAAG
g17877.t2-FORWARD	AATAGTAGTCTCGTCAACTTCTTCGC
g17877.t2-REVERSE	TCGCAGCCTCTCGTTCTCTTC
g17997.t1-FORWARD	GCCGAGTCAAGGAGGAGGTTC
g17997.t1-REVERSE	ATGGTGGAGGAGCTGGTTCTTC
g18359.t1-FORWARD	CCTGCGGTTTCAGATGTTAATTTGG
g18359.t1-REVERSE	CTTGCTGGTGTGATTCTATGTTCTTC
g23282.t1-FORWARD	TTCAGAAGAGGAGAGAAGCATTTGC
g23282.t1-REVERSE	GACGCCGAGAGGAGGATGATAG
g25236.t1-FORWARD	CAACACCAAGAAACTGCTACAAATCG
g25236.t1-REVERSE	GCACTGCCATGACTAGGAATGAC
g25942.t1-FORWARD	GATCCACCTATCAATCACCATCTTCC
g25942.t1-REVERSE	ACTAACGCTTGCTTGTCTCTTCG
g26841.t1-FORWARD	GAACCGACTAGGCATTGCTCTAAG

g26841.t1-REVERSE	GAGCCTTGATGGTGTCTGTATTGG
g29399.t1-FORWARD	CAGGGAGGCGAAGACGGATG
g29399.t1-REVERSE	GGGCTCGGGCATGGACTTG
g30373.t1-FORWARD	TGATCCAGATCGTTGGGAATTTGC
g30373.t1-REVERSE	TGTGATGGTTGTTGTGGTTGACTATG
g31168.t1-FORWARD	TGCGACGGAAGTAACGGTAGC
g31168.t1-REVERSE	TTGGGTTGCCGATGGATTTGATG
g31755.t1-FORWARD	CAGGAAGATGGATAAGGAGCAATGG
g31755.t1-REVERSE	TGAATGGCTGTGAACTGGTTTACG
g33546.t1-FORWARD	TCTTCGTCGTCCTCATCTTCACC
g33546.t1-REVERSE	GTCCGTCGCAGAATCTTCCAC
g34334.t1-FORWARD	CTTGCCGAGCCCATTGACTTC
g34334.t1-REVERSE	CTTATTTGCCCTTCGCCGTATCC
g36487.t1-FORWARD	CGGTCTACGAAGGCACTGATAAG
g36487.t1-REVERSE	TCATCTCCGTCTCCATCTCCATC
g36767.t1-FORWARD	GGTAGCCGAGTTGTCTGAGGAG
g36767.t1-REVERSE	CCCACCAAATCGTCGCACTG
g38782.t1-FORWARD	GGAAGATTGGCACAGGTAGATGG
g38782.t1-REVERSE	GGAGATTTGCTGCTGCTTGTTATAC
g38809.t1-FORWARD	ATGATGGCGTTTCTTCACAAATTCG
g38809.t1-REVERSE	GGCAGGTGGCGTCTCTTCC
g39393.t1-FORWARD	TCCTCGACCCTTTGCAGTTCTC
g39393.t1-REVERSE	TTCTTGAATCCGTAGGTGTTGAGTTG
g40203.t1-FORWARD	GAAGAGATTTCACCCAGAACAATGC
g40203.t1-REVERSE	CACGAGGAAGGAGGCATAAGATTG
g40444.t1-FORWARD	TTGGTGTGACAGAGTATGTTGAAGAG
g40444.t1-REVERSE	GCCGCTGTTTCCACGAGAAC
